# Commentary: “Multimodality advanced cardiovascular and molecular imaging for early detection and monitoring of cancer therapy-associated cardiotoxicity and the role of artificial intelligence and big data”

**DOI:** 10.3389/fcvm.2023.982028

**Published:** 2023-02-27

**Authors:** Louise Y. Sun, Gift Echefu, Krishna Doshi, Michelle L. Roberts, Abdulaziz Hamid, Richard K. Cheng, Jessica Olson, Sherry-Ann Brown

**Affiliations:** ^1^Division of Cardiothoracic Anesthesiology, Department of Anesthesiology, Perioperative and Pain Medicine, Stanford University School of Medicine, Stanford, CA, United States; ^2^Department of Internal Medicine, Baton Rouge General Medical Center, Baton Rouge, LA, United States; ^3^Department of Internal Medicine, Advocate Lutheran General Hospital, Park Ridge, IL, United States; ^4^Division of Cardiovascular Medicine, Department of Medicine, Medical College of Wisconsin, Milwaukee, WI, United States; ^5^Medical College of Wisconsin, Milwaukee, WI, United States; ^6^Cardio-Oncology Program, Division of Cardiology, University of Washington, Seattle, WA, United States; ^7^Institute for Health and Equity, Medical College of Wisconsin, Milwaukee, WI, United States; ^8^Cardio-Oncology Program, Division of Cardiovascular Medicine, Medical College of Wisconsin, Milwaukee, WI, United States; ^9^Department of Cardiovascular Diseases, Mayo Clinic, Rochester, MN, United States

**Keywords:** cardio-oncology, big data, artificial intelligence, bioinformatics, cancer therapy

## Introduction

As novel oncologic treatments are developed, clinicians are challenged with the diagnosis and prevention of adverse cardiac events in cancer patients. Conventional imaging techniques such as echocardiography (Echo) and magnetic resonance imaging (MRI) can predict adverse cardiac outcomes by assessing wall motion and left and right ventricular function but cannot detect subclinical cardiotoxicity. Advanced imaging techniques employ innovative technology to conventional imaging modalities to enhance early cardiotoxicity detection and thus promote time management (1). Application of late gadolinium enhancement and hyperpolarization to MRI and molecular targeted radiotracers are examples of such strides to improve cardiac imaging specificity. This commentary builds upon the recent study reviewing the latest research on cardiac imaging in the surveillance of chemotherapy-related cardiotoxicity in cancer survivors and discusses the potential of artificial intelligence (AI) in this space (2). Furthermore, it will highlight knowledge gaps in cardio-oncologic care and detail the hope for the clinical deployment of AI to improve health outcomes (2).

Applying AI and big data to cardiovascular imaging could revolutionize the investigation of cardiotoxicity by identifying patterns and signals beyond the capability of existing imaging techniques. For the effective deployment of AI, the availability of comprehensive and multimodality data is crucial. However, there are several challenges to using big data and AI for clinical care and research that prevail in cardio-oncology. For example, cardiotoxicity can present with disparate patterns and a spectrum of outcomes affecting almost any aspect of the cardiovascular system. In addition, cancer is a heterogeneous group of conditions with a wide range of disease patterns, expected outcomes, and responses to therapy.

AI can be attuned to address these issues. However, the algorithms may not be readily interpretable or explainable to the end users. Therefore, avenues are needed to develop more efficient ways to accelerate the deployment of diagnostic modalities for predicting and early detection of cardiovascular toxicities related to oncological therapies. Incorporating AI into routine cardio-oncology care could address knowledge gaps, increase access, address racial/ethnic inequalities, and shift the paradigm toward prevention *via* earlier identification of cases (3–6). Integrating AI applications into the clinical workflow is a crucial step toward maximizing the potential benefit of established AI algorithms. In a study by Lui et al., four key elements for the successful deployment of AI into the clinical workflow are: (1) **AI orchestration**, with data imputation and machine learning (ML), to create robust models that can be integrated into the workflow, (2) **Testing of AI applications** retrospectively and prospectively on internal data to confirm applicability before deployment, (3) **Processing steps** to address speed, protection for data in cloud-based systems, and hardware configuration, (4) **Active involvement and collaboration** of trained personnel, including cardio-oncologists, oncologists, data scientists, software engineers, radiologists, IT specialists, internal review boards, and legal partners (7).

## Discussion

Early diagnosis is possible by assessing global longitudinal strain on an Echo (1, 4). Although not currently employed in clinical practice, ongoing investigations indicate that fast strain encoded measures obtained from cardiac MRI are sensitive and highly accurate in identifying subclinical cardiotoxicity (4). Cardiovascular MRI can also detect late gadolinium enhancement, a marker for myocardial injury or scarring. However, it does not always provide information about the type, cause, or extent of damage to the heart. Targeted imaging modalities are needed to improve cardiac imaging specificity. For example, the iodine-123-labeled metaiodobenzylguanidine nuclear scintigraphy imaging marker is useful in the detection of anthracycline-associated cardiotoxicity (6). However, this marker can also be non-specific and is routinely used for the detection of adrenaline secreting tumors (6). Furthermore, several radiotracers have shown utility in the detection of cardiotoxicity, specifically atherosclerotic disease (8). Molecularly targeted probes against CD4 and CD8, or increased uptake of somatostatin receptor 2 in implicated vessels detectable by the positron emission tomography (PET) tracer ^68^Ga-DOTATATE has been shown to predict plaque rupture (8). ^68^Ga-FAPI is another PET radiotracer tracer targeting fibroblast activating protein which has been shown as a potential early marker of immune checkpoint inhibitor-induced myocarditis. However, more investigations are needed to understand their specific value in clinical environments (9). Echo and MRI are the imaging modalities recommended by the European Society of Medical Oncology and the American Society for Medical Oncology for assessing oncolytic therapy-mediated cardiomyopathy (10). Through the detection of alterations in global longitudinal strain, Echo and MRI can assess wall motion abnormalities, left and right ventricular function, and are also able to detect early indicators of cardiotoxicity.

MRI effectively evaluates immune therapy-mediated cardiotoxicity, including pericarditis and myocarditis, by assessing myocardial edema and extracellular volumes. Patients receiving immunotherapies are at an increased risk of myocarditis. Echo and MRI can evaluate and detect early signs of cell infiltration and myocarditis. Compared to echo, the versatility of MRI allows for additional tissue characterization using T1, T2, extracellular volume measurement, and delayed gadolinium enhancement assessment (4). Normal myocardial cells do not enhance with gadolinum, as MRI-DGE relies on the differences in tissue conditions and underlying pathology (11). It can detect myocardial damage or scarring but lacks specificity in delineating early end-stage fibrosis (11–13). Conditions associated with myocardial edema such as myocarditis or acute myocardial infarction which can be reversible exhibit gadolinium delays indistinguishable from chronic infarction and fibrosis.

Nuclear scans, like a multigated acquisition scan, lack the sensitivity and availability of echo and MRI, but their clinical diagnostic utility is currently being explored. Emerging techniques are employing molecularly targeted radiotracers to elucidate pathomechanisms in cardiotoxicity. In cardiovascular MRI applications where sensitivity is an issue, hyperpolarization can significantly boost the signal to overcome these barriers. Hyperpolarized magnetic resonance with radiotracers allows unparalleled detection of tissue metabolism through real-time assessment of substrate uptake and enzyme transformation *in vivo*.

## Knowledge gaps in cardio-oncology care

AI application in clinical practice is not without limitations. Noteworthy are the knowledge gaps that limit the general applicability of big data in clinical practice. The field of medicine has been historically plagued by health inequities, including poor access to healthcare and representation affecting ethnic/racial minorities, as well as individuals of low socioeconomic status. Consequently, the available data for integration into AI algorithms will likely reflect this information bias and further healthcare disenfranchisement.

In cardio-oncology, pitfalls in the scarcity of dedicated practicing specialists and limited awareness regarding chemotherapy-related cardiotoxicity among general cardiologists directly impact survivorship. To ensure the full impact of the exponential growth in developing novel anticancer therapies, clinician awareness of the risk factors and populations at risk is paramount. African American race and female sex have been reported as risk factors in the development of cardiotoxicity related to immune checkpoint inhibitors, including pembrolizumab (14). Therefore, it is imperative to leverage novel predictive technologies to ensure that these subgroups are referred early for cardio-oncology care. Sadler et al. (15) conducted a comprehensive, collaborative study and program development across the Florida chapters of the American College of Cardiology and American College of Clinical Oncology to assess physician knowledge gaps around chemotherapy-related cardiotoxicities among the two specialties. They found that only 14% (23/163) of oncologists and 16% (22/134) of cardiologists were comfortable managing cardio-oncology patients; more than half of cardiologists reported below-average knowledge of cardio-oncology care and cardio-oncology services were available to less than half of respondents in each group (15).

Existing health disparities also limit the general applicability of AI-based big data in clinical practice. There is well-documented evidence of a disproportionately higher incidence of fatal cancer and heart disease among ethnic/racial minorities and underserved communities (16, 17). Minorities are less likely to undergo interventions for cancer prevention, screening, early detection, and therapy, including surveillance and management of treatment-associated cardiotoxicity (18). This exists in the backdrop of limited access to cardio-oncology service, affordability and impact of low socioeconomic status, geographic distance, and transportation barriers (19–21). Furthermore, ethnic/racial minorities are underrepresented in pivotal clinical trials for cancer therapeutics, leading to difficulty generalizing these results in these subpopulations after FDA approval (18, 22–24).

Cardiovascular disease causes long-term morbidity and mortality in cancer survivors. Over the last 40 years, cancer survival rates in the United States have increased, with an estimated 18.1 million survivors as of January 2022 (25). By 2040, the population is predicted to have 26 million cancer survivors, with the majority at risk of cardiotoxicity (26). Of the clinical manifestations of cancer treatment-related cardiotoxicities, cardiomyopathy or left ventricular systolic dysfunction is the most debilitating. Guidelines directing prevention, screening, detection, and monitoring for cancer treatment-related cardiotoxicities are limited but are growing in number and breadth of pathologies considered in the past several years (10, 27, 28). Currently, Echo is most frequently used for assessing left ventricular function. Biochemical markers (particularly troponin and brain natriuretic peptide) are emerging approaches to identifying individuals at risk of cardiotoxicity (4). These modalities may be limited by operator expertise and the quality and availability of images. AI and big data can address these limitations by integrating all available sources of information into a user-friendly platform and delivering consistent, efficient, and accurate precision-based predictive and diagnostic information.

Ultimately, the knowledge gaps are potential bottlenecks curtailing the full potential and robustness of AI use in clinical practice.

### The promise of big data and AI in cardio-oncology care

Medicine is traditionally reactive in nature. For hundreds of years, clinicians have followed patients to diagnose and treat disease after it has developed. In recent decades, the focus has shifted to proactive management and prevention on a population health level. Clinicians and data scientists work toward predicting who is likely to develop disease and implement preventive measures in these high-risk groups. In those who have already developed disease, the focus is placed on predicting complications of therapy and developing strategies to mitigate risks.

**Figure 1:**
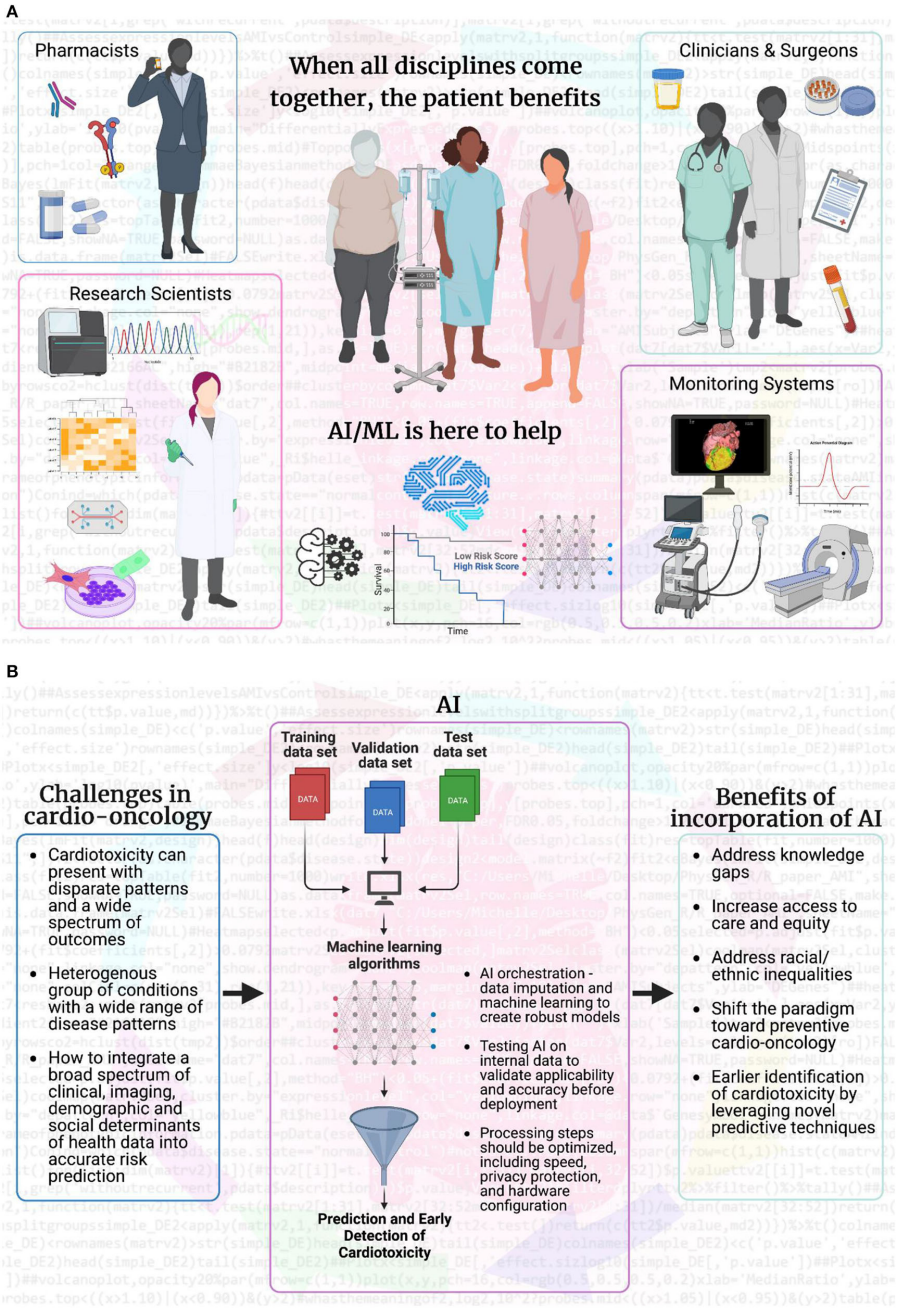
Artificial intelligence and machine learning bridge gaps to make patient care more effective. **(A)** AI and ML fuel scientific research by analyzing big data from multiple sources: individual patients, healthcare professionals (pharmacists, research scientists, clinicians, oncologists, surgeons, cardiologists, and advanced cardiovascular imaging experts), and imaging/monitoring systems (ultrasound, MRI, CT, PET, and electrophysiology). Clinicians and surgeons are at the forefront of health discussions and can access patient information and fresh or banked samples. AI/ML can analyze imaging beyond visual capabilities and integrate results into the decision-making process for pharmacists and scientists. Pharmacists can incorporate information on patient over-the-counter, pain management, and adherence to prescription medication into AI/ML systems. When combined with patient outcomes, AI/ML can achieve the goal of maximum treatment with minimal adverse effects, even years after treatment is complete. AI/ML can also guide research scientists working behind enemy (disease) lines, to inform experimental design for collected samples, prospectively or retrospectively. AI/ML combines and integrates multiple sources of information with high computing and cloud power to provide a higher level of quality care for each individual patient. **(B)** The overall flow of how AI/ML can aid in addressing the current challenges facing the field of cardio-oncology and how the incorporation of AI/ML will be beneficial for predictive and early detection, as well as preventive care in cardio-oncology. Created with BioRender.com.

The shift toward proactive care is possible with the advent of big data and state-of-the-art bioinformatics techniques (29). Modern data scientists are equipped with the ability to harness a variety of longitudinal data housed in electronic health records, medical images, and molecular profiles. These data include administrative, demographic, clinical profiles, laboratory, imaging, omics, and outcomes (30). Integration of this data enables prediction and surveillance of disease progression and treatment-related complications and allows timely, personalized interventions to improve response to treatment and survival. AI and modern advances in computational technology helps to unlock disease patterns and seamlessly integrate complex datasets in real-time to enable proactivity in clinical care in an unbiased and computationally efficient manner (29). Integrating AI algorithms into the clinical workflow will help clinicians unlock disease patterns that are not readily visible to the human eye. On a broader scale, AI could enhance the clinician's ability to predict, prevent, and treat the cardiovascular sequelae of cancer therapy in a more timely and effective manner (Figure 1).

### Clinical deployment of AI

We must acknowledge and address limitations to overcome the challenges of integrating AI into clinical settings. Algorithms must be based on large datasets representing all ethnic/racial and sociodemographic groups within the population served. Algorithms trained on small, local datasets may not necessarily be applicable in other practice settings. The utilization of historical datasets in algorithm training and the possibility that adopting a new algorithm would alter clinical practice hinder the model's efficiency during future rollout (31).

Support from healthcare professionals using AI algorithms is also critical. ML may be viewed by clinicians as uninterpretable “black boxes,” which precludes their readiness for uptake (32). Active education of key stakeholders, including clinicians, administrators, policymakers, patients, and the general public, with a clear demonstration of the value added by AI and big data, is necessary to encourage adoption. Furthermore, there is significant evidence that collaboration among data scientists, engineers, ethicists, and information technology professionals is crucial to the implementation success of next-generation technology in clinical settings. Using human induced pluripotent stem cells (hiPSCs) in research laboratories and biobanks for modeling cardiotoxicity in specific cardiac cell types in culture required tremendous buy-in for patient accrual (31, 32). The microfluidic chip environment can be used in broad molecular applications (cell signaling, electrophysiology, “-omics” analysis) but requires trust from biomedical research teams to be incorporated into standard laboratory practice (33). AI can potentially add spatial and temporal analytics of imaging modalities to the cellular and molecular levels of information, but it also requires the same level of trust and collaboration (34). These data can be incorporated so that therapies can be adapted, and the patient receives the greatest benefit while disease-free tissues are preserved (33). When all disciplines come together, the patient benefits (Figure 1).

## Conclusion

AI-enabled tools have the potential to revolutionize the cardio-oncology landscape by improving and personalizing diagnostic and treatment plans in a timely and comprehensive manner, using integrated data from all available sources in real-time. This innovative approach will improve patient outcomes by personalizing the individual care experience and making targeted recommendations based on patient profiles. By mobilizing multidisciplinary teams and incorporating the perspectives and needs of all collaborators, AI-based innovations can overcome implementation challenges and improve outcomes and long-term survival for cancer therapy-treated patients.

## Author contributions

LYS and SAB led the development of the commentary manuscript. LYS, GE, and KD wrote sections of the manuscript. MR, AH, and RKC generated the figure. LYS, MR, JO, and SAB oversaw the writing, review, and revision of the manuscript. All authors contributed to the manuscript review, revision, and approved the submitted version.
